# Can micronutrient requirements be met by diets from sustainable sources: outcomes of dietary modelling studies using diet optimization

**DOI:** 10.1080/07853890.2024.2389295

**Published:** 2024-08-11

**Authors:** Ursula M. Leonard, Mairead E. Kiely

**Affiliations:** a Cork Centre for Vitamin D and Nutrition Research, School of Food and Nutritional Sciences, University College Cork, Cork, Ireland; b INFANT Research Centre, Ireland, University College Cork, Cork, Ireland

**Keywords:** Sustainable diets, plant-based diets, nutrition, micronutrients, bioavailability, diet optimization, review

## Abstract

**Background:**

Consumption of diets from sustainable sources is required for planetary health, however, large sections of the population, including females of reproductive age and children, will be at risk of not meeting their micronutrient (MN) requirements in a complete transition to plant-based foods. Constrained diet optimization methods use mathematical programming to construct diets that meet predefined parameters and may contribute towards modelling dietary solutions that meet nutritional and planetary targets.

**Objective:**

Review the evidence from diet optimization studies proposing solutions to ensure MN availability in the context of a transition to diets from sustainable sources.

**Approach:**

Narrative review focusing on literature published over the last five years.

**Results:**

Dietary modelling using diet optimization can design a range of omnivorous and plant-based diets that meet individual MN requirements, have reduced environmental impacts, and minimize deviation from culturally acceptable dietary practices. Using data from large-scale dietary surveys, diet optimization can support development of food-based dietary guidelines; identify limiting MNs in a particular context or a conflict between constraints e.g. nutrition and environment; explore food-based strategies to increase nutrient supply, such as fortification; and support trial design. Methods used and outcomes reported are sources of variability. Individual-level dietary data and MN requirements for population sub-groups such as females of reproductive age and children are important requirements. Although maintaining iron and zinc intakes are regularly reported to present challenges in diets from sustainable sources, few studies have considered bioavailability, which reduces with increased dietary phytate. These and other data gaps including acceptability and affordability must be addressed to improve the applicability of modelling outcomes in population recommendations.

**Conclusions:**

Dietary modelling using diet optimization can be useful in the design of more sustainable diets that meet MN requirements, however, translation of outcomes into dietary intervention studies is required to test real-world application and adoption into dietary guidelines.

## KEY MESSAGES

1. A widespread shift towards plant-based diets could increase the risk of inadequate intakes of micronutrients typically sourced in foods of animal origin, particularly among females of reproductive age and children.
2. Modelling of dietary intake data collected among individuals using techniques such as diet optimization could be helpful in guiding recommendations and intervention strategies; translation into intervention studies is required.
3. Representative data in different population groups and settings, with methods incorporated to address data gaps on bioavailability of micronutrients, food fortification, and affordability, will improve the applicability of modelling outcomes.

## Introduction

Food systems require positive transformation to meet health and environmental objectives that are central to the Sustainable Development Goals (SDGs) and Paris Agreement [1–3]. A widespread shift in food consumption towards diets from sustainable sources is an integral component of this transformation. Defined by the Food and Agriculture Organization of the United Nations (FAO), these are diets with ‘*low environmental impacts, which contribute to food and nutrition security and to healthy life … are protective and respectful of biodiversity and ecosystems, culturally acceptable, accessible, economically fair … nutritionally adequate, safe and healthy*’ [4]. The four domains of health and nutrition, environmental protection, affordability, and society are key pillars of sustainable food systems [4,5].

Food-based dietary guidelines (FBDGs) provide public health advice on foods, food groups and dietary patterns which are designed to supply the nutrients required to prevent chronic diseases and promote overall health [6]. National FBDGs have lower environmental footprints compared to actual dietary patterns in high income countries [7]. However, when adherence to national FBDGs in 85 countries were modelled, Springmann et al. [8] reported that a third would fail to meet SDG #3.4 (reduce premature deaths from non-communicable diseases (NCDs) by one third), and most fall short of sustainability targets e.g. limit global warming to <2 °C, which highlights potential gaps in population recommendations. Some countries have started to introduce environmental considerations into FBDGs e.g. Denmark [9]. Others promote a plant-based dietary pattern through aggregation of food groups e.g. the Canadian ‘*Food Guide Snapshot*’ which displays three food groups on a plate including vegetables/fruits, protein foods, and whole grains [10]. The latter, which does not provide quantitative recommendations, has been questioned on its supply of calcium and vitamin D [11].

Published in 2019 by the EAT-Lancet Commission, the *Planetary Health Diet* is rich in wholegrains, vegetables, fruits, legumes, nuts, and unsaturated vegetable oils, with moderate amounts of fish and poultry, and low amounts of red meat and dairy [12]. It was proposed that adapting the *Planetary Health Diet* to local circumstances could help meet sustainability targets and reduce premature mortality by 34% [8]. To date, the *Planetary Health Diet* has not been investigated in dietary intervention studies. A deeper analysis of the assumptions and data supporting the diet has suggested that females of reproductive age and children, who are at higher risk of micronutrient (MN) malnutrition, especially in low-income settings, will struggle to meet their MN requirements, which are higher relative to their energy consumption [13]. Our analysis of the published literature [14] reported that lower intakes and status of MNs of public health concern are a potential outcome of dietary changes to reduce environmental impacts and that inadequate research data are available to quantify this impact among vulnerable life-stage groups. This narrative review aims to further consider the potential impact on MN availability in the context of a transition to plant-based diets, focusing on data from dietary modelling experiments using diet optimization techniques.

## At risk micronutrients of public health concern

Vitamins and minerals, collectively known as MNs, are required in small amounts daily (micrograms or milligrams) and play a critical role in supporting growth, development and maintaining health throughout the life-course [15]. Due to lower energy intakes and higher requirements during growth and development, females of reproductive age and children are at higher risk of inadequate MN intakes and status than the male adult population, especially during pregnancy, lactation and periods of rapid growth [15,16]. In low income settings, women and children can be disproportionately affected by food shortages and lack of nutrition security [17].

During pregnancy, low MN status increases the risk of adverse perinatal outcomes, including hypertensive disorders of pregnancy, preterm delivery and low birth weight [16,18–20]. This is also a critical period for the developing brain, where MNs such as iron, iodine, zinc, copper, selenium, vitamin A, choline and folate are required for synapse formation, myelination and other developmental processes, particularly in the third trimester of pregnancy and early neonatal period [20–22]. Iron deficiency is the most common MN deficiency globally, occurring with anemia in its most severe form; infants born to iron deficient mothers are at higher risk of anemia and low iron stores, which are linked to altered brain structure, reduced cognitive function, academic performance, work capacity, and behavior [23]. Low maternal supply of iodine impacts the production of fetal thyroid hormones that are key for neurological development and severe deficiency can result in iodine deficiency disorders [16,20,22]. Iodine requirements increase by 50% during pregnancy and insufficient intakes are common in Europe [24]. Mild-to-moderate maternal deficiency in the first trimester, when the fetus is completely dependent on maternal iodine, can lead to reduced cognitive performance [20]. In children and adolescents, lasting physical and developmental consequences can arise from inadequate supply of MNs needed for musculoskeletal formation and growth [25–27]. Calcium and/or vitamin D deficiency causes nutritional rickets [25,27] and reduces attainment of peak bone mass, increasing the risk of fractures and osteoporosis in later life [26].

Representative data on the prevalence of MN deficiencies are lacking, due to low prioritization or advocacy for MN status surveillance in public health policies, cost, expertise required to implement such surveys, and access to adequate facilities including laboratories with skilled staff and equipment [28]. Variation in analytical methods for MN biomarker analysis can make inter-laboratory comparisons difficult, however, recommendations from expert working groups assist in assessment of MN status in individuals, including choice of biomarker, consideration of inflammation, and reference values for deficiency [28]. A recent study attempted to estimate the prevalence of MN deficiencies globally using existing nationally representative MN status data (iron, zinc, folate, vitamins A, B_12_ and D) from 22 countries [29]. The authors estimated that one in two children (aged 6-59 months) and two-thirds of non-pregnant females (aged 15-49 years) are deficient in at least one MN [29]. MN deficiencies were not confined to low-income countries e.g. an estimated 55% of females in the United Kingdom (UK) are deficient in at least one MN, and 21% of females and 31% of children in the UK were iron deficient (serum ferritin <15 µg/L and <12 µg/L, respectively) [29]. The prevalence of two or more MN deficiencies occurring simultaneously varied, however, some countries such as India have a high prevalence in both children (40%) and females (57%). In Europe, Cashman et al. [30] reported that 13% of individuals had very low vitamin D status, with a 25-hydroxyvitamin D (25(OH)D) <30 nmol/L (12 ng/ml), the level at which risk of metabolic bone disease increases. This study reported that individuals with dark skin pigmentation were over 3-times more likely to have very low vitamin D status, although data were relatively few. In a maternal-infant cohort in Ireland, 49% of women with dark complexion had a 25(OH)*D* < 30 nmol/L at 15 weeks of gestation, versus 17% of the predominantly pale-skinned cohort [31]. Among umbilical cord sera, 46% were < 30 nmol/L, illustrating the intergenerational impact of MN status [32].

Many at risk MNs are primarily sourced and/or most bioavailable from animal sourced foods and if these sources are not available to those who need them, suitable alternatives are required [33]. Notably, prevention of vitamin A deficiency, the leading preventable cause of blindness globally [16] and a major predictor of infection-related mortality in young children, has been demonstrated effectively using biofortification of crops such as sweet potato or cassava, ensuring adequate supply of pro-vitamin A carotenoids [34,35]. Biofortified iron crops have demonstrated effectiveness in increasing iron status, and studies have found that biofortified wheat can provide bioavailable zinc [34].

In the transition to more sustainable diets, the challenge will be to ensure adequate supply and accessibility of MNs in the food system for everyone, within environmental constraints. *In silico* modelling experiments are a useful approach to considering these challenges.

## Dietary modelling of sustainable diets

The evidence base assessing the transition to diets from sustainable sources is largely theoretical, mainly based on secondary analyses of observational data, and there are many open questions regarding dietary composition and its impacts on nutritional status [14,36–38]. Dietary modelling uses existing dietary data to explore strategies such as measuring the impact of adherence to published dietary guidelines or specific dietary patterns, as well as measuring the impact of dietary substitutions targeting specific foods or food groups, which can range from substitutions of ruminant meats with monogastric meats or plant-based foods, to partial or complete replacement of animal-source foods with plant-based foods [14,37,39,40].

This review focuses on outcomes from studies using the dietary modelling approach based on constrained diet optimization (diet optimization hereafter), which uses various forms of mathematical programming to obtain an ‘optimal diet’ based on a set of predefined parameters [40–43]. The model optimizes an objective function (minimized or maximized), which is commonly set to minimize the deviation from the baseline diet, represented by existing dietary intake data, most appropriately from nationally representative surveys or large-scale cohort studies. The aim of minimizing the deviation from baseline is to create a culturally acceptable solution. The decision variables are the foods available for selection, that are combined to produce the ‘optimal diet’, along with information such as current consumption quantities, nutritional composition, environmental impact, price, etc. Constraints are set to define the domain where solutions can be found, which can include lower and/or upper bounds. Examples of constraints include MN intake target ranges based on dietary reference values (DRVs), food intake targets based on FBDGs, environmental indicators, limits on cost, and limits on acceptable changes from current consumption amounts e.g. within 5^th^ – 90^th^ percentile. Diet optimization is successful when a combination of the decision variables meets the objective function and satisfies all constraints [40–43].

Diet optimization has been used to answer questions on dietary recommendations and nutrient supply, complementary feeding among infants, and food security [41,42]. Early studies focused mainly on identifying nutritionally adequate diets within defined cost constraints, and from around 2010 onwards, started including environmental considerations [40–43]. More recently, diet optimization has been used to propose MN adequate diets with lower environmental impacts in several European countries [44–65], India [66,67], China [68,69], Iran [70,71], Brazil [72], Lebanon [73], Tunisia [74], and globally [75,76]. Diet optimization requires expertise and collaboration between nutritional scientists and modelers. There is inherent variability in the approaches taken, arising from different data sources, objective functions and constraint settings, unique to the research question being addressed.

## Applications of diet optimization for developing healthy diets from sustainable sources

Diet optimization is commonly used to model an optimized diet based on mean intakes in a population [46,48,53,54,65,67,70,71,73–75]. For example, Broekema et al. [46] based decision variables on dietary intakes from the Dutch National Food Consumption Survey. With the objective function of minimizing deviation from the baseline dietary pattern, diets were optimized to meet MN intakes at the level of the Dutch Population Reference Intake (PRI), whilst limiting greenhouse gas emissions (GHGEs). The PRI is the level of a nutrient estimated to meet the needs of almost all (97.5%) individuals in a population [15]. Twelve scenarios included three different Intergovernmental Panel on Climate Change (IPCC)-informed GHGE reduction targets and a range of omnivorous and plant-based dietary patterns. The model successfully optimized all 12 scenarios, resulting in reductions in beef, pork, poultry, cheese, butter, and snacks, and increases in legumes, fish and shellfish (where applicable), nuts, vegetables, and soy products. The use of 12 scenarios demonstrates the variation in dietary intakes that are compatible with a set of constraints, however, the vegan scenario depended on a fortified soy drink to meet calcium and vitamin D intakes (consumption increased from 9 to 532 g/day). Constraints can be added to foods or food groups to tailor outcomes to a population or setting e.g. an acceptability scenario, which limited dietary changes to 33-150% of baseline intakes, resulted in a more diverse diet. Limitations of this approach include the inability to account for the distribution of food intakes in a population arising from using the mean.

Diet optimization can also propose solutions to food and nutrition security in different settings. For example, Damerau et al. [67] optimized diets to explore the potential of India’s agricultural sector to produce nutritious foods. Using household-level dietary intake data (per capita weighted average) in decision variables, diets were optimized to maximize the supply of MNs whilst constraining GHGEs, water, and land use. Two scenarios were included, based on current and future per capita resources (land and water). Both scenarios led to an increase in intakes of the 20 included MNs and an estimated 14-27% reduction in premature deaths. However, vitamin B_12_ and total iron (future scenario) intakes remained below the Estimated Average Requirement (EAR) of the World Health Organization (WHO). As the EAR is the level of a nutrient estimated to meet the needs of 50% of the population, this indicates a supply risk. Using food supply data, Chaudhary and Krishna [75] optimized diets to remain as close to baseline as possible for 152 countries. Constraints were placed on nutrition, acceptability, and environmental impact. A strength of this study was the inclusion of absolute limits for GHGEs, water, land, nitrogen, and phosphorous, estimated to keep planetary boundaries within a safe operating space [77]. They demonstrated that this was possible, for all individuals in all countries, while providing an adequate supply of MNs according to the WHO recommended intakes. The deviation from the baseline diet was considerable for all countries, but reasons differed e.g. reduced animal-sourced foods in North America and increased energy availability in Africa. Intakes of pulses, fruits, and vegetables led to increased environmental footprints for low- and middle-income countries. Limitations of this approach include the lack of information on food distribution within a household.

Individual dietary intakes can also be used in diet optimization studies [44,45,76]. By setting the objective function to minimize the deviation from baseline, this finds the closest optimal combination of foods that satisfies all constraints for each individual. In France, two studies optimized nationally representative dietary intakes with constraints on nutrition, health, acceptability, and GHGEs (30% reduction) [44,45]. Gazan et al. [44] set all constraints at the level of the individual, meaning that all diets achieved a 30% reduction in GHGEs from baseline by reducing meat, increasing plant-based products with no impact on dairy. Rocabois et al. [45] took a different approach by setting the constraint to reduce GHGEs by 30% at the population level, which allocated GHGE mitigation efforts according to baseline GHGEs. Participants were categorized into three GHGE reduction groups: high ‘–45%’, medium ‘–30%’, low ‘–14%’ and one group that could accommodate an increase ‘+11%’. While the direction of change in dietary intakes was similar for most foods, the magnitude differed e.g. ruminant meat reduced by 55 g/day in the ‘–45%’ group but only 9 g/day in ‘+11%’ group. The trend was similar for other animal-sourced foods. Thus, both studies achieved the same GHGE target with different impacts on individual diets.

## Opportunities for diet optimization

### Inform the development of food based dietary guidelines

The European Food Safety Authority (EFSA) recommends a stepwise approach for developing FBDGs: 1) identify diet-health relationships; 2) identify country-specific diet-disease relationships; 3) identify nutrients of public health concern; 4) identify food groups for inclusion based on contribution to nutrients; 5) identify food consumption patterns; 6) test the nutrient composition of FBDGs; and 7) develop graphical representations [78]. Diet optimization offers a novel and systematic method to support FBDG development by identifying combinations of foods/food groups which remain close to the observed consumption in a population whilst meeting DRVs and reducing the risk of NCDs. This was demonstrated by Mariotti et al. [79] in their development of healthy eating patterns for FBDGs in France. Using mean intakes from nationally representative survey data, diets were optimized to minimize the deviation from the baseline diet, minimize (e.g. red meat) or maximize (e.g. vegetables) the intake of food groups associated with NCD risk, and minimize exposure to contaminants. Criteria in the objective function and constraints were applied in a step-by-step approach to assess their compatibility; constraints included MN intake ranges (French PRI and adequate intake, and EFSA Tolerable Upper Intake Level (UL)), food groups associated with risk of NCDs, dietary habits for acceptability, and food contaminants. The first scenario included MN intake and food group targets, which were achieved, but the diet deviated considerably from baseline, containing only 11 of the 32 available food groups, with large amounts of vegetables and wholegrain starches. When dietary habits were introduced, adaptations were needed to achieve a solution, including identification of substitutable food groups e.g. whole grains and refined grains; removal of upper limits for some food groups; and reduction in intake targets for vitamin D and iron (females). Of note, more adaptations were required for females to achieve MN targets, due to lower energy intakes.

Further, the possibility of including environmental sustainability considerations within the model offers a comprehensive method for the design of more sustainable FBDGs. Using mean intakes from nationally representative data from the Netherlands, Brink et al. [65] used diet optimization as the basis for developing their sustainable FBDGs. The objective function was set to minimize deviation from baseline, with constraints on nutrition (Dutch PRI and EFSA UL), health, environmental impact, and acceptability. Health constraints included recommendations in current guidelines e.g. 200 g fruit/day. Environmental impact constraints included limits on animal-sourced foods e.g. 50^th^ percentile of current intake for females. Acceptability constraints included upper limits for some food groups based on baseline intakes to avoid unrealistic changes e.g. 135 g legumes/week. Modelled intakes of fruit, legumes, fish, and shellfish were similar across groups, whereas vegetable intakes for females aged 31–50 were triple that of males of the same age, and red meat ranged from 8 g in females 19–50 to 70 g in males over 70. Expert judgment was used to translate the optimized diet intakes into practical quantitative guidelines for 18 age and gender groups, with some additional versions created (e.g. pregnancy), and reviewed by dieticians and consumers.

Diet optimization may provide more acceptable solutions for sustainable FBDGs than other dietary modelling methods. Using mean intakes from nationally representative data, Nordman et al. [54] optimized Danish diets to minimize deviation from the baseline diet, i.e. the departure score, with constraints on nutrition, health, and GHGEs. Initially, they optimized a diet to reduce GHGEs, which fell short of some MN targets and had a departure score of 16%. When nutritional considerations were added, the optimized diet deviated further from baseline, with a departure score of 38%. Intakes of ruminant meat, soft drinks, alcohol, animal fats, cheese, discretionary foods, and pork (particularly processed) reduced. Dairy intake remained similar, and intakes of nuts, bread, eggs, and fish increased, with redistribution to fish with lower GHGEs (e.g. shrimp to mackerel). A big driver of the departure score was a large increase in nuts from baseline (+230%). When the increased iron requirements for premenopausal females were considered, the departure score increased to 73%, driven by increases in wholegrains, potatoes, pork offal, and eggs. The results of this optimization study were compared to a previous modelling analysis based on the EAT-Lancet’s *Planetary Health Diet* [80] which was used to inform the Danish FBDGs [9]. The Danish *Planetary Health Diet* did not meet vitamin D or iodine recommended intakes, and had a departure score of 169%, driven by increases in legumes, nuts, seeds, and green vegetables [80]. Both the Danish optimized and *Planetary Health Diet* achieved the same reduction in GHGEs, however, the optimized diet included more culturally acceptable foods e.g. pork, eggs, and milk.

### Identify at-risk micronutrients in a population or setting

Diet optimization can support both individual requirements and consider local contexts, such as food supply, which are important elements for consideration in the transition to plant-based sustainable diets [81]. One method is the identification of limiting MNs, known as active constraints, which are the MNs that are included in the optimized diet (MN intake of diet) at the same level defined in the constraint [41]. Table 1 provides a summary of the limiting MNs reported by optimization studies. Iron, zinc, calcium, and sodium were regularly reported as active constraints. This indicates that these MNs influenced the combination of decision variables more than others in that population, potentially due to baseline intakes or conflicts with other model constraints. Notably, only six of these studies accounted for potential differences in the bioavailability of iron and zinc.

**Table 1.** Summary of micronutrient constraints identified as limiting (included in the optimized diet at the level defined in the constraint) in diet optimization studies.

	Minerals								Vitamins						
Study	Fe	Na	Zn	Ca	I	Se	K	Mg	A	C	B_12_	D	B_6_	B_2_	B_9_
Kesse-Guyot et al. [56]	✔^1^	✔	✔^1^	–	–	–	–	–	–	✔	–		–	–	–
Kesse-Guyot et al. [59]	✔^1^	✔	✔^1^	–	–	–	–	–	–	–	–		–	–	–
Salome et al. [60]	✔^1^	✔	✔^1^	–	✔	–	–	–	✔	✔	✔		✔	–	–
Fouillet et al. [61]	✔^1^	✔	-^1^	✔	✔	–	–	–	✔	–	✔		–	✔	–
Dussiot et al. [62]	✔^1^	✔	✔^1^	–	✔	–	✔	–	✔	✔	–		–	–	–
Vieux et al. [63]	✔^2^		✔^2^	✔	✔	–		✔	–	–	–	✔	–	–	–
Dussiot et al. [64]	-^1^	✔	-^1^	–	✔	–	–	–	✔	✔	–		–	–	–
Sobhani et al. [71]	–	✔	–	✔				–	✔	✔	✔		✔	–	✔
Eustachio Colombo et al. [53]	✔^3^	✔	–	✔	✔	✔	–	–	✔	–	–	✔	–	–	–
Nordman et al. [54]	✔		–	✔	–	✔	–	–	–	–	–		–	–	–
Ferrari et al. [55]	✔		✔	✔							–				
Verly-Jr et al. [72]	–		–	✔			✔	–	–	–	–		–	–	–
Tompa et al. [58]	✔	✔	✔	✔			✔	–	–	–	✔	✔	–	–	–
Total (12)	10	9	7	8	6	2	3	1	6	5	4	3	2	1	1

Key: ✔, limiting micronutrient; -, non-limiting micronutrient included as a constraint.

^1^Bioavaiable intakes.

^2^The iron constraint assumed high iron losses through menstruation for females under 50 years, and the zinc constraint was reported to be appropriate for the levels of phytate.

^3^Intake based on increased DRV for iron for plant-based diets.

Abbreviations: Ca, calcium; Fe, iron; I, iodine; K, potassium; Mg, magnesium; Na, sodium; Se, selenium; Zn, zinc.

In some cases [60–62], an upper bound constraint for vitamin D was set at 100 µg/day for safety, however the lower bound was set to 0 µg/day. Although vitamin D deficiency prevalence is widespread [30], it is usually omitted from the constraints or included as a fraction of the requirement [58,63] due to synthesis of cholecalciferol in skin through sun exposure. The limited availability of vitamin D in the food supply impacts outcomes in multiple ways, which was explored by Bruins et al. [47] when optimizing the diets of Dutch adults. With the objective function of minimizing deviation from the baseline dietary pattern, four scenarios with varying constraints were assessed, including a daily target of 13.4 µg of vitamin D. A diet optimized to meet MNs at the level of the EFSA PRI resulted in a 178% increase in GHGEs (10.7 kg CO_2_ equivalents/day) and required an energy intake of almost 4000 kcal/day. When constraining energy to 2000 kcal/day, a maximum of 9.6 µg/day of vitamin D could be achieved, but resulted in a 71% increase in GHGEs (6.6 kg CO_2_ eq/day). The inclusion of additional vitamin D fortified foods in decision variables (whole grain bread, semi-skimmed milk, and oil) met the vitamin D target, but resulted in an 8% increase in GHGEs. Finally, the addition of a GHGE constraint resulted in a 9% reduction in GHGEs, however, meeting the vitamin D target depended heavily on fortified foods requiring a shift in consumption practices.

### Assess the impact of strategies to support MN supply

Food fortification, the addition of MNs to foods during processing, can target key MNs that are at risk in the transition to more sustainable diets, and be superior to supplementation due to lower personal responsibility in compliance and cost effectiveness [82,83]. Grasso et al. [49] used diet optimization to model three dietary scenarios with varying levels of fortification of plant-based alternatives (meat, dairy, and fish), bread, and oil. Scenario 1 included foods currently available in the Dutch National Food Consumption Survey including fortified margarine (vitamins A and D), dairy alternatives (vitamins B_12_, D and riboflavin), and meat alternatives (iron, calcium, and vitamins B_6_ and B_12_); Scenario 2 included plant-based alternatives fortified at 30% of the Codex Alimentarius nutrient reference values, including vitamins B_3_, B_6_, B_12_, D, riboflavin, selenium, iron, zinc, calcium, and iodine; and Scenario 3 included additional fortification of all breads and oils at 15% of the Codex Alimentarius nutrient reference values (same MNs as point 2). All optimized diets met MNs at the level of the Dutch PRI, while reducing GHGE based on an IPCC-defined target for GHGEs in 2030. The inclusion of fortified foods resulted in a lower deviation from the baseline diet measured by Euclidean distance; a lower proportion of animal protein from total protein (Scenario 1:2:3 was 33%:23%:19% for females and 20%:16%:10% for males); and lower use of water and land resources. Fortification policies differ between countries and regions, hence, diet optimization can help inform solutions suited to a specific setting [82]. The inclusion of MN supplementation is rarely considered, but can impact outcomes; Walker et al. [76] reported that individual diets including fish and supplements resulted in lower dietary GHGEs compared to vegetarian and vegan diets, driven by nutrients such as vitamin D and omega 3.

### Support the development of varied diets from sustainable sources

Diet optimization can be used to explore varying dietary patterns, providing flexible or personalized solutions for diet modification, e.g. interrogating the optimal ratio of plant-to-animal protein. Fouillet et al. [61] optimized diets in France to maximize health and minimize deviation from the baseline diet, with the aim of finding the optimal proportion of plant-protein [61]. Constraints were placed on nutrients, including bioavailable iron and zinc, and acceptability. A range of solutions with between 25-70% plant-protein met all constraints, with an inverse relationship between the environmental impact and % plant-protein. Optimized diets had no red meat and increased intakes of fruits, vegetables, legumes, nuts, and milk. Milk and seafood were the last animal-sourced foods to be removed as the proportion of plant-protein increased. When plant-protein went >80%, diets did not meet targets for iodine, vitamin B_12_ (males), bioavailable iron (females), and calcium. All modelled diets ranging from 35-65% plant protein deviated from baseline diets, demonstrating the gap between current and healthy diets, regardless of the plant-to-animal protein ratio.

Another analysis from this research group assessed the impact of reducing ruminant meat in French diets using two scenarios [64]. First, the diet was optimized to maximize health, with nutritional and acceptability constraints. Although not directly targeted, ruminant meat was removed completely in diets optimized for males and reduced by 93% in diets optimized for females. Poultry increased in place of ruminant and other red and processed meats. Intakes of fruits, vegetables, wholegrains and eggs also increased, and dairy and fish remained stable. In the second scenario, ruminant meat was reduced in 10% increments from baseline. To maintain a culturally acceptable diet, the objective functions were set to maximize health and minimize the deviation from the baseline diet, with constraints on nutrients and acceptability. A solution was found at each level, however, there were considerable dietary changes driven by the nutritional constraints from the initial 10% reduction step. Total meat reduced initially but remained constant thereafter at ∼100 g/day, mainly consisting of poultry. Diet composition remained quite consistent between the 50-100% reduction steps, indicating that the health criteria were achieved. Although environmental indicators were not considered in the model constraints, ruminant-meat free diets (100% reduction) resulted in less GHGEs (-29%) and land use (-36%) but required more water (+47%) and energy (+5%). Inclusion of environmental constraints within the model will refine the domain where solutions can be found, and provides important insights into the impact of reducing consumption of a nutrient-rich food group such as ruminant meat.

## Challenges for designing diets from sustainable sources

### Stratification of vulnerable population groups

Meeting the MN needs of women and children can be challenging due to a combination of high MN requirements and lower energy intakes. Consideration of age and gender through stratification of dietary data is essential to uncover discrepancies. Ferrari et al. [55] optimized Italian diets to minimize GHGEs, with nutritional, health, and acceptability constraints. Optimized diets had approximately 50% lower GHGEs, however, only a solution was found for males. The higher iron requirement for females (17 mg/day) was not compatible with all constraints, the maximum intake possible was similar to males at 11.8 mg/day. A limiting factor was a constraint set to keep red meat within 10 to 30 g/day. In China, Song et al. [68] optimized diets to minimize GHGEs, water, and ecological footprint, with constraints on nutrition and acceptability. Of the 18 age and gender groups (8-80 years) included, the higher MN requirements of adolescent groups aged 14-17 years resulted in the smallest GHGE reduction magnitudes. Studies of requirements during pregnancy and lactation are scarce [65].

Basing the foods in decision variables on individual-level dietary intakes from nationally representative surveys [44–49,53–63,65,68–74] rather than household-level [50,66,67,84] or national-level supply [51,75] data can support individual variation by reducing the risk of masking low individual intakes due to data aggregation. Furthermore, using individual gender- and age-based requirements as lower bounds in MN constraints will ensure that outcomes are compatible with all population groups.

### Acceptability of optimized diets

A common approach to address accessibility and acceptability is to minimize the deviation from the baseline dietary pattern in the objective function [44–49,53,54,56,57,59,65,66,72–75]. As more than one combination of decision variables can satisfy the model constraints, the model is forced to find the closest possible optimal combination of foods [40,41]. Linear programming favors large changes in few foods; a non-linear method such as quadratic programming [48,54,59,65,73] is recommended by some authors as it favors small changes in many foods by minimizing the sum of the squared differences in intakes, deemed to provide more realistic results. However, the nature of this objective function can result in foods with low consumption not increasing as one might expect. For example, the intake of legumes in the Danish population assessed by Nordman et al. [44] was 1 g/day and did not increase in the optimized diet. As legumes are a food group recommended in sustainable healthy dietary patterns [4,66] and may be of interest when designing such diets, a food-based constraint could be added to the model to meet the desired intake. Many studies place acceptability constraints on the permitted dietary changes, which can avoid extreme changes in single foods or food groups. Examples include restricting food group changes to within the 5^th^ and 95^th^ percentile [55] or below the 95^th^ percentile [56,59] of current intakes. Grouping similar foods together to allow interchanges without penalization can produce more practical results e.g. fresh, dried and processed fruits [60,62,64]. Although these measures may enhance the likelihood of producing acceptable optimized diets, translation of outcomes into dietary intervention studies is required to assess acceptability.

### Bioavailability of foods and dietary patterns

The bioavailability of some MNs e.g. iron and zinc, differ based on food sources and dietary patterns, where absorption is enhanced by compounds such as meat tissue and ascorbic acid, and inhibited by compounds such as phytate [85,86]. Traditional absorption factors for omnivorous diets may overestimate iron bioavailability in plant-based diets; however, diet-dependent absorption equations, which consider the enhancers and inhibitors in the individual’s diet, can provide a more accurate bioavailable intake estimate [86,87].

Most studies do not consider potential differences in bioavailability, however examples of both constant absorption factors [53,57] and diet-derived factors [56,59–62] are reported. Regardless of environmental considerations, Dussiot et al. [85] found that bioavailable iron and zinc were the most difficult MNs to meet when optimizing French diets to comply with healthy dietary patterns. Zinc was the most limiting constraint in male diets and iron in female diets, however, both MNs were limiting in all populations, likely impacted by similar dietary sources and dependance on phytate. Compared to baseline, optimized diets contained more fruits, vegetables, wholegrains, and red meat (females), and less processed meat and soft drinks (males). Total meat intake remained similar for males and increased for females with high iron requirements. To understand the influence of the limiting constraints on diet composition, the targets for bioavailable iron and zinc were relaxed. This resulted in a reduction in meat and red meat, and an increase in grains and wholegrains. Of note, the relaxed optimized diet was estimated to increase the prevalence of iron-deficiency anemia from 0.2% to 5.0% in adults (0.1% to 7.0% in females) but reduce the overall risk of chronic disease and anemia by 18%.

Similarly, using data from the NutriNet-Santé cohort in France, Kesse-Guyot et al. [59] found that meat intake remained stable in a diet optimized with 50% less GHGEs, but there was a redistribution of beef and lamb to pork, driven by bioavailable iron and zinc. In the diet of males, wholegrains were reduced almost completely (2 g/day) due to the trade-off between phytate and bioavailable zinc, which was reversed when the zinc constraint was lowered. The relatively high environmental impacts of meat make it a target for reduction when environmental considerations are included in diet optimization [88]. These studies demonstrate the potential pitfalls for iron and zinc intakes if bioavailability is not considered.

### Cost implications

Affordability is a key domain of sustainable diets, however, cost is often omitted from diet optimization studies. Healthier diets are reported to be more expensive, which could increase socioeconomic inequalities, thus, consideration should be encouraged to prevent disparities [89]. A challenge is the volatile nature of foods which has seen large price spikes over the past number of decades, impacted recently by the COVID-19 pandemic and the Russia-Ukraine conflict [90]. However, including cost as a constraint can provide insights into the current affordability implications. For example, Chaudhary and Krishna [66] optimized diets for 35 states in India with constraints on nutrition, environmental impact, acceptability, and cost. When cost was limited to current expenditure levels, most states could not meet vitamins A, B_12_, E, riboflavin, pantothenic acid, or calcium (some states). Fruit, vegetables, and dairy were limited by the cost constraint, with large reductions in dairy impacting vitamin B_12_. When the constraint was increased to 1.5 times the current expenditure levels, all states could meet individual MN recommendations. Affordable foods with low environmental impacts were identified for each MN e.g. beans for iron, zinc, niacin, potassium, and calcium, and carrots for vitamins A and E. Within a country, lower-income groups may be disproportionally impacted; to consume a diet optimized for the Brazilian population, the relative cost difference for the lowest income level was almost twice the average, and five times higher than the highest income level [72]. However, Reynolds et al. [50] demonstrated that diets could be optimized for different income levels (quintiles) in the UK to meet MNs at the level of the UK RNI, reduce GHGEs (35-38%), and reduce cost (18p-47p/day). Dietary changes were similar across income quintiles; increased intakes of fruits, vegetables, starchy foods, and seafood, and reductions in animal-sourced foods, non-alcoholic beverages, and foods high in fat and sugar.

Although environmental impacts were not considered, Johnson-Down et al. [91] demonstrated how diet optimization can be used to optimize the diets of minority groups who have a high prevalence of food insecurity. The diets of a Canadian Indigenous population, First Nations individuals living on reserves, were optimized to minimize deviation from the baseline diet, with constraints on nutrition, acceptability, and cost. When constraining nutrients only, diet cost increased for females and there were considerable dietary changes including 500% more diet drinks, milks, fruit, meat alternatives and fish, and large reductions in oils and fats, beef, pork, poultry, eggs, and soup. When acceptability and cost was constrained, the diet was deemed to be more acceptable and had a lower cost, however, intakes of calcium, potassium, vitamin D, phosphorous (females) and magnesium and vitamin A (males) fell below the target. Of note, all nutrients other than calcium and vitamin D improved from baseline. This analysis demonstrates potential trade-offs between constraints, which may disproportionately impact food insecure contexts.

### Keeping pace with advances in product development

The food environment landscape is changing rapidly, and plant-based alternative products are now commonly available as substitutes for animal-sourced foods [36]. The list of foods included in decision variables is often restricted to foods present in the data source, which may not contain these substitute foods when using older sources [53]. The process of converting reported food quantities into decision variables typically requires aggregation, by converting branded to generic foods [30], and grouping similar foods together [24,25], which may lose some variation [53]. This challenge is compounded by the wide variation in the composition and fortification of plant-based substitutes identified in food audits [92,93]. Of 132 plant-based meat analogs on the market in Australia, only 12% were fortified with iron, zinc, and vitamin B_12_ [92]. In the UK, an analysis of 136 plant-based dairy products revealed gaps in fortification; 43% of milk-, 36% of yogurt-, and 87% of cheese-substitutes contained no calcium; and 96% of milk- and all yogurt- and cheese-substitutes contained no iodine [93]. A fortification standard would help address this variability [94].

## Conclusions

The domains of health and nutrition are central to sustainable healthy diets [4,81]. Prevalent MN deficiencies, particularly among females of reproductive age and children, could be exacerbated by a widespread shift among these groups towards plant-based diets, which needs to be considered carefully before dietary recommendations are formulated [13,14,29]. Outcomes of diet optimization modelling, particularly models based on dietary intakes collected on an individual basis, can generate a range of omnivorous and plant-based dietary proposals that are likely to be more compatible with reduced environmental impacts while meeting nutrient recommendations and minimizing the deviation from culturally acceptable dietary patterns. Although the outcomes of diet optimization have been incorporated into FBDGs, they are yet to be tested in an experimental setting. A large proportion of the published data exists for high-income countries, although these countries have a higher potential for meaningful change, more representation of middle- and lower-income countries is required, including consideration for population sub-groups. Data gaps are many, including food composition data that considers the fortification of foods, especially ‘substitute’ products such as plant-based protein sources and dairy substitutes. Bioavailability is also a priority question for MNs such as iron and zinc. These MNs, along with calcium, are consistently identified as limiting MNs, meaning that they are the most difficult to meet when transiting to plant-based sustainable diets. These nutritional considerations and other sustainability-related domains such as affordability need to be addressed to facilitate more comprehensive analyses.

## Data Availability

Data sharing is not applicable to this article as no new data were created or analyzed in this study.
